# Public Health and Online MICE Technology During the COVID-19 Pandemic: The Role of Health Beliefs and Technology Innovation

**DOI:** 10.3389/fpubh.2021.756987

**Published:** 2021-10-01

**Authors:** Jinge Yao, Qiwei Pang, Binyuan Zhang, Lu Wang, Yiling Huang

**Affiliations:** ^1^Department of Economics, Sejong University, Seoul, South Korea; ^2^College of Arts and Physical Education, Myongji University, Yongin, South Korea

**Keywords:** COVID-19, social distancing, online MICE, public health, health beliefs, technology innovation

## Abstract

The traditional meetings, incentives, conferences, and exhibitions (MICE) industry has been hit hard by social distancing regulations introduced to combat the COVID-19 pandemic, with concerns about pandemic risks and personal hygiene increasing the demand for online MICE technology. With the introduction of innovative new technologies to the MICE industry, it is important to study the psychology of online MICE attendees, particularly the factors affecting their behavioral intention to adopt online MICE technology during the pandemic. This study investigates the attitudes toward attending online MICE since the start of the epidemic based on the health belief model (HBM) and innovation diffusion theory (IDT). A total of 439 valid questionnaires were collected in China and used for structural equation modeling. The results show that the perceived safety threat, the comparative advantage, trialability, and outcome expectations positively impact the attendees' attitudes. Moreover, this study finds that attitude completely mediates the impact of perceived safety threat, comparative advantages, trialability, and outcome expectation on behavioral intention to attend online MICE events. These findings theoretically enrich the understanding of online MICE technology, the HBM, and the IDT and offer managerial implications for MICE organizers and exhibitors.

## Introduction

The 2019 new coronavirus (COVID-19) emerged in December 2019 in Wuhan, China, and rapidly spread around the world. The World Health Organization (WHO) reported that, as of December 7, 2020, there were 66,243,918 confirmed COVID-19 cases and 1,528,984 deaths worldwide (1). The WHO has strongly advised avoiding contact with others and maintaining social distancing due to the significant risk of COVID-19 transmission (2). This global healthcare and economic crisis has negatively affected trade and associated economic activities, including production, transportation, storage, and distribution (3).

The meetings, incentives, conferences, and exhibitions (MICE) industry is currently one of the fastest-growing tourism sectors both globally and domestically China (4–6). In addition to the important industry ties that are created during MICE events, the MICE industry has major economic benefits for host countries and communities, including promoting economic growth, encouraging exports, and fostering cultural and creative exhibitions (7). As such, in many countries, the MICE industry is considered vital to strengthening national competitiveness. According to the International Congress and Convention Association (ICCA), the number of international association meetings doubled every 10 years between 1963 and 2010, followed by a 26% increase between 2010 and 2019. However, in 2020, the MICE industry was severely hit by quarantine regulations and worldwide border closures in most countries due to the global COVID-19 pandemic (8). According to a report by the ICCA, the total number of MICE events held in 2020 was 8,409, a decrease of 36.55%. The top three MICE-hosting regions include Europe (4,706; 55.96% of the world convention market), Asia (1,501; 17.85%), and North America (980; 11.65%). These three regions account for 85.47% of the total, and the majority of MICE were postponed 3,714 (41%), 2,503 (30%) of these are online MICE. In 2020, the number of attendees to the exhibition was 4.057 million, of which the postponed (1,558,075; 38%) and online MICE (1,509,460; 37%) attendees comprised a similar-sized proportion of the total number of attendees (9, 10). This indicates that while the number of online MICE attendees was lower than the number of postponed attendees, the number of participants of online MICE was higher.

With the development of information technology, many MICE events now offer an online interactive experience (11). In addition, due to time, space, cost, and energy constraints, many companies cannot hold effective or satisfying offline MICE events, and this has been exacerbated by the COVID-19 pandemic. In this context, online MICE events can offer a powerful information distribution function that allows participating companies to overcome these constraints. Transitioning from offline to online MICE events can also reduce resource consumption, environmental pollution, and infection risks caused by traffic, the printing of publicity materials, and the setting up of booths (12). More importantly, the contactless nature of online MICE events may meet the attendees' mental and physical expectations, encouraging their participation and ensuring their safety from the public safety crisis (13). Therefore, during the COVID-19 pandemic, to further promote the orderly development of the MICE industry; promote emerging technologies, deep MICE industry integration, and innovation; and launch online MICE businesses, it is important to break through the traditional limitations. The exhibition of the industry provides product promotion, brand exhibition, technology exchange, and trade negotiation via an online communication platform to further promote the digital and online transformation and development of the exhibition industry, so that customers can enjoy the MICE events at home and explore business opportunities.

With the increasing popularity of online MICE, research in this area has received significant attention from various perspectives, including the solution of online MICE holding mode (14), user requirements for online exhibitions (12), and the sustainable development of the online MICE industry (15). Although most governments and companies are aware of the benefits of online MICE events and are willing to invest in online infrastructure, their success ultimately depends on the experience of the attendees and whether they are willing to use online MICE technology (16). However, despite the rapid investment in and development of the online MICE industry, less research attention has been paid to objectively evaluating the online MICE industry from the perspective of attendee acceptance during the COVID-19 pandemic. In particular, the level of acceptance of online MICE technology among attendees and whether online MICE events provide the same or a better experience than offline events need to be investigated.

In order to address these issues, the present paper develops a theoretical model to identify the attendees' behavioral intention to participate in online MICE events during the COVID-19 pandemic. The innovation diffusion theory (IDT) (17) and the health belief model (HBM) (18) are introduced as the theoretical foundation for the conceptual framework of the present study. The HBM was chosen because it can be used to analyze and identify potential psychological factors associated with the use of online MICE, such as perceived safety threats and comparative advantages, while the IDT was employed based on the trialability and outcome expectations associated with MICE technology and because it considers the communication about or sharing of an innovation within a social system through communication channels. These theories have been considered appropriate for studying the behavioral intention associated with technology and for use in COVID-19 research (19, 20).

The remainder of this study is structured as follows. We present the theoretical foundation and hypotheses in section Literature Review and Hypothesis Development, while section Methodology describes the research methodology and data collection. Section Results and Discussion describes the data analysis, hypothesis testing, and results. Finally, section Conclusions summarizes the contributions, limitations, and recommendations for future research.

## Literature Review and Hypothesis Development

This study examines the extent to which the behavioral intention of online MICE technology is influenced by the health beliefs, attitudes, and cues to action for attendees during the COVID-19 pandemic. This section provides a summary of the two main theories used to establish the model for the present study, the HBM and the IDT, and presents the associated hypotheses.

### Health Belief Model

The HBM was first proposed in the early 1950's by social psychologists (21), and has since been widely used in the health behavior industry to better understand health education and interventions (22). This model recognizes that personal health beliefs and the effects of those beliefs on attitudes toward preventive activity may be the first in a series of events leading to health promotion. Based on this, health educators can improve their risk communication based on a solid understanding of the psychological mechanisms (23). Though the HBM was created to understand patient practices in relation to specific diseases or their willingness to have early checkups for these diseases, this study proposes that the model can be used to explain the safety behavior associated with online MICE because this behavior can be viewed as a way to prevent or reduce the probability of contracting a disease (24, 25). According to the HBM, health beliefs play a significant role in preventive health behavior, and the ways of knowing and acting are founded on subjective schemata (26, 27). According to the HBM, perception variables such as the perceived safety threat, outcome expectations (a composite score based on perceived barriers and advantages), and self-efficacy can predict health or protection behavior (28). These beliefs are thought to be part of the cognitive mediation process (29). However, little research has been undertaken to investigate the effects of health attitudes on health risk avoidance behavior in the MICE context. This is despite the fact that there is undeniable proof that travel and tourism can hasten the spread of infection (30) and that ignoring the importance of protective health habits can lead to new outbreaks in local communities (31).

### Innovation Diffusion Theory

The IDT was created to explain why people choose to accept or reject a new technology based on their beliefs (32). While online meetings are not new, they are being utilized more frequently now than in the past, and to individuals participating in them for the first time, they often appear to be new technology (33). According to the IDT, comparative advantages, compatibility, complexity, observability, and trialability are all factors that influence whether or not an innovation is adopted (34, 35). A comparative advantage represents the superiority of a current innovation to similar previous innovations, while compatibility is the degree to which an invention is believed or perceived to be consistent with the adopter's values, needs, and previous experiences (36). Complexity is the degree to which an innovation is viewed or regarded as difficult to use (34), while observability is the degree to which the outcomes of the innovation are evident and trialability is the degree to which an innovation can be implemented in stages (37). The most widely identified explanations for a consumer's intention to adopt new technology have been comparative advantages and trialability (38). Therefore, this study focuses on examining the influence of these two factors on attendees' behavioral intention to participate in online MICE events.

**Figure 1:**
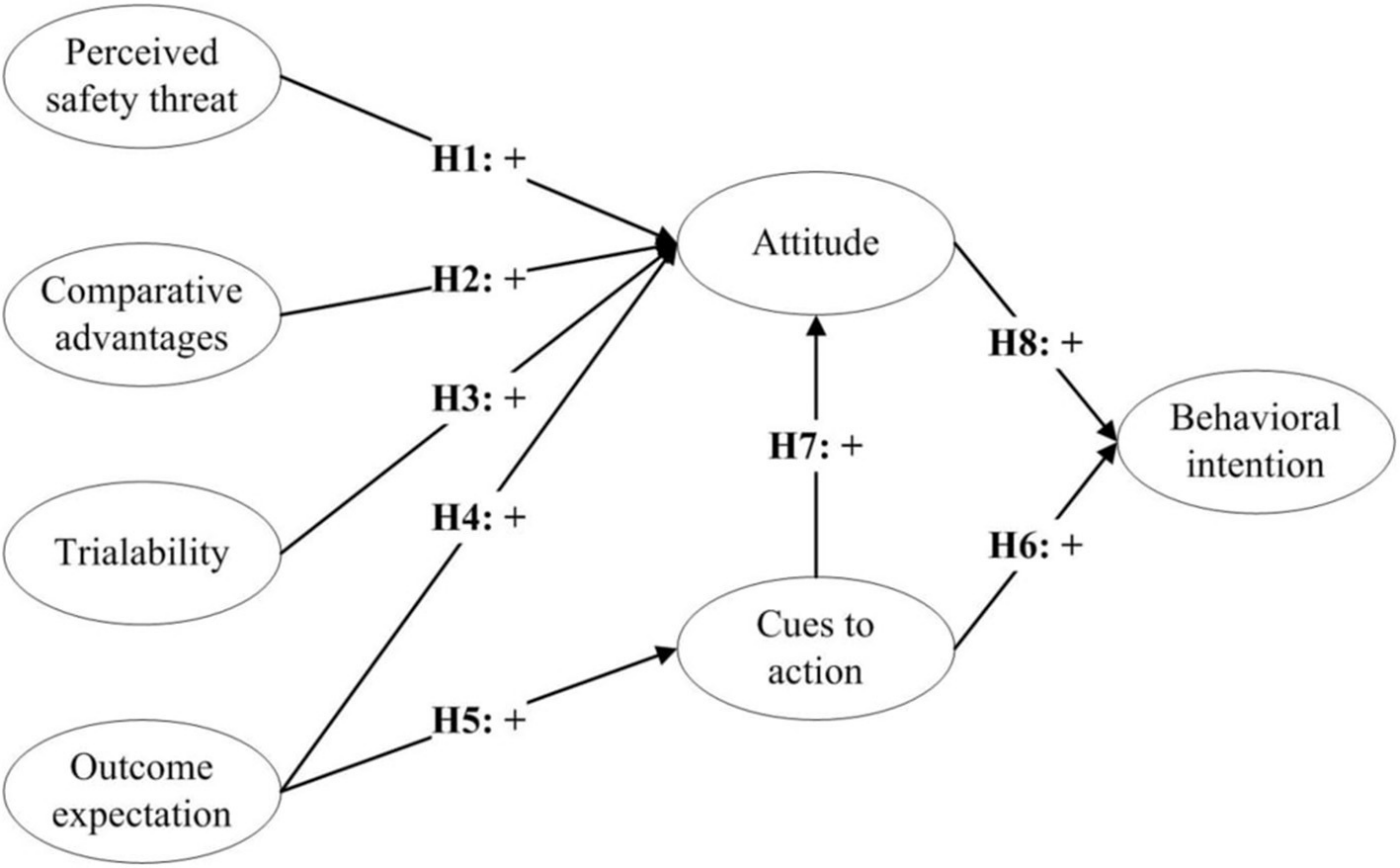
Conceptual framework.

Based on these two theories, this study offers a modified theoretical model that incorporates the HBM and IDT to examine the antecedents influencing attendees' behavioral intention to attend online MICE. This model is presented in Figure 1.

### Hypothesis Development

#### Perceived Safety Threat

Attendees' subjective assessment of the negative effects (i.e., the severity and vulnerability) of a lack of safety behavior is characterized as the perceived safety threat (39). The perceived safety threat of COVID-19 has led to a dramatic increase in emotional disorders, cognition stress, and arousal problems (40). As a result, if they sense a threat to their safety from the virus, the attendees will exhibit an extremely careful attitude to avoid negative consequences. If the perceived advantages surpass the perceived costs, an attendee is more likely to demonstrate a positive attitude. Thus, we offer the following hypothesis:

H1. The perceived safety threat of COVID-19 has a positive effect on attendees' attitudes toward attending online MICE events during the pandemic.

#### Comparative Advantage and Trialability

One of the most important concepts in the IDT is the comparative advantage. A comparative advantage is defined as the degree to which an invention is seen to provide greater benefits than its predecessor (41, 42). The greater the apparent competitive advantage, the more quickly an innovation will be adopted (43). Trialability refers to the extent to which the public can experience an innovation before deciding whether or not to accept it. For those who can observe an innovation in use by others or who can use it themselves to learn, an invention that can be trialed reduces doubt among potential users (44). As a result, trialability is defined in this study as the attendees' acceptability toward the use of an online MICE system, which influences their behavior. Previous research has shown that there is a link between comparative advantage, trialability, and consumer attitudes (45–48). Therefore, the following hypotheses are proposed:

H2. A comparative advantage has a positive effect on attendees' attitudes toward attending online MICE events.
H3. Trialability has a positive effect on attendees' attitudes toward attending online MICE events.

#### Outcome Expectations and Cues to Action

The concept of outcome expectance refers to the belief that an action will have some consequences (49). The attitude toward a certain object or action may be influenced by expectations for that action or object (50). The motivation for a certain action is based on the expectation that they can predict the occurrence of the target state (51). That is, a person chooses certain behaviors based on their expected results. Action cues such as surrounding information and experience can be developed into related incentive models to form motivations (52). Therefore, Hsu et al. (50) argued that motivation can be shaped by manipulating the cues that define the motivational value of an individual's consequences of their actions. In their study, cues to action were used as a motivational variable and its intermediary effect between expectations and action was verified. Therefore, this research proposes the following hypotheses:

H4. The outcome expectations for COVID-19 have a positive effect on attendees' attitudes toward attending online MICE events during the pandemic.
H5. The outcome expectations for COVID-19 have a positive effect on cues to action.

#### Effect of Cues to Action on Attendees' Attitudes and Behavioral Intention to Attend Online MICE Events

Cues to action refer to stimuli that affect the intention of an individual to use a certain service (31), and these can originate from social influence, personal experience, or potential change possibilities (53). The HBM posits that people need a cue to action to motivate their readiness to engage in a health behavior (54). During the COVID-19 pandemic, if the participants in offline MICE events can have COVID-19, the risk associated with participating in an offline MICE will increase, which will have a negative impact on attendees' intention to participate in offline MICE events and a positive impact on the behavioral intention of online MICE technology. In addition, strong action cues will have a positive impact on behavior. For example, Tsai et al. (55) reported that effective nursing education as a health promotion strategy can improve the behavior of nursing students and prevent the spread of COVID-19 both locally and globally. As such, the benefits of following positive cues to action will encourage consumers to develop positive attitudes such as trust in a technology (32), and it will also facilitate purchase intention or behavioral intention. Thus, this study establishes the following hypotheses:

H6. Cues to action have a positive impact on attendees' attitudes toward attending online MICE events.
H7. Cues to action have a positive impact on attendees' behavioral intention to attend online MICE events.

#### Effect of Attitude on Attendees' Behavioral Intention to Attend Online MICE Events

In this study, we utilized the definition of attitude proposed by Zhao et al. (56) and Hu et al. (57), referring to individual feelings regarding specific behaviors, while behavioral intention is defined as the desire to perform a specific behavior. This definition is used for consumer behavior research because of its strong predictive ability (58). According to previous research, the more strongly that consumers want to buy opinion products, the more likely they are to purchase those products in the future (59). With regard to MICE events, previous research has also shown that if an individual has a positive attitude toward online MICE technology, they are likely to adopt this technology (60). Thus, this study examines the relationship between attitude and the behavioral intention to adopt online MICE technology and proposes the following hypothesis:

H8. Attitude has a positive impact on attendees' behavioral intention to attend online MICE events.

## Methodology

### Survey Design and Data Collection

This study used an anonymous cross-sectional survey of current users (adopters) and non-users (non-adopters) of online MICE technology to test the hypotheses. The information obtained by cross-sectional survey is collected at a given point in time (61). The ‘point in time’ that data was collected in this study was during the COVID-19 pandemic. The non-adopters included in the survey are considered potential users of the online MICE technology.

The survey was conducted in China because exhibitors increasingly prefer to hold events in Asia–Pacific countries such as China, rather than the traditionally strong markets in Europe and the United States (10). By consulting experts and scholars in the MICE field, we compiled a questionnaire for online MICE technology.

The survey consisted of three sections. In order to ensure that all respondents (especially non-adopters) were fully aware of how online MICE operated, Section 1 introduced the working mechanisms and main features of online MICE technology; this description was illustrated with relevant images. Section 2 asked questions about the perceptions, attitudes, and intention to adopt online MICE technology, while Sect ion 3 collected demographic information about the respondents, including their gender, age, education, and income.

To collect a representative sample, this study conducted the online survey using Wenjuanxing, a professional data science company (62). To ensure that the participants were motivated to complete the survey, they were told they would receive a small digital gift from the researchers at the end of the survey. From May 28 to June 19, the link for the questionnaire was posted to the WeChat group of company employees and the WeChat group of MICE attendees and 505 responses were received. After eliminating surveys in which the screening question was answered incorrectly (a question that asked the respondent to answer “strongly disagree” was added in the second section) or that were not sufficiently complete, 439 valid questionnaires were used for subsequent analysis.

### Measurement Items

The survey contained 26 items designed to measure seven variables: perceived safety threat, comparative advantages, trialability, outcome expectations, attitude, cues to action, and behavioral intention (Table 1). Each variable was measured using items from the following sources: perceived safety threat from Yuen et al. (31), comparative advantages from Wang et al. (45) and Shih and Fang (47), trialability from Wang et al. (45), outcome expectations from Yuen et al. (32), cues to action from (63), attitudes from Shih and Fang (47) and (63), and behavioral intention from Agag and El-Masry (35). Many studies have shown that seven-point Likert scale show higher reliability than any other number of options (64, 65). The items were evaluated using a seven-point Likert scale ranging from 1 (“strongly disagree”) to 7 (“strongly agree”).

**Table 1:** Scale development.

**Construct**	**Measurement items**	**References**
Perceived safety threat (PST)	*Strongly disagree (1)/Strongly agree (7)*	(31 )
	PST1. My chance of contracting COVID-19 is low if I use online MICE.	
	PST2. Because of my physical health, I am more likely to be infected by COVID-19 if I use offline MICE.	
	PST3. If I contracted COVID-19, it would compromise my personal financial security.	
	PST4. The thought of suffering COVID-19 fills me with dread.	
Comparative advantages (CAD)	*Strongly disagree (1)/Strongly agree (7)*	(45, 47)
	CAD1. Using online MICE is more efficient than using offline MICE during the COVID-19 pandemic period.	
	CAD2. Using online MICE would save time.	
	CAD3. Using online MICE would improve my overall attendance experience compared to offline MICE during the COVID-19 pandemic period.	
	CAD4. Using online MICE would be advantageous compared to offline MICE during the COVID-19 pandemic period.	
Trialability (TRI)	*Strongly disagree (1)/Strongly agree (7)*	(45)
	TRI1. I feel it is easy to try out online MICE.	
	TRI2. I know where I can go to try out various functions of online MICE.	
	TRI3. I am permitted to try out online MICE over a sufficiently long period.	
	TRI4. I am able to experiment with online MICE when necessary.	
Outcome expectations (OEX)	*Strongly disagree (1)/Strongly agree (7)*	(32)
	OEX1. I use online MICE to interact with exhibitors and staff members during the COVID-19 pandemic period.	
	OEX2. Using online MICE is truly a joy.	
	OEX3. Compared to the cost, I think I will receive good value while using online MICE during the COVID-19 pandemic period.	
	OEX4. Using online MICE will compensate for what I miss during the COVID-19 pandemic period.	
Cues to action (CTA)	*Strongly disagree (1)/Strongly agree (1)*	(63)
	CTA1. My colleagues and partners will support me if I use online MICE during the COVID-19 pandemic.	
	CTA2. I will use online MICE during the COVID-19 pandemic if more people are using online MICE.	
	CTA3. Overall, I am encouraged by the authority to use online MICE during the COVID-19 pandemic.	
	*Strongly disagree (1)/Strongly agree (7)*	
Attitude (ATT)	ATT1. I feel using online MICE is a wise idea.	(47) (63)
	ATT2. I like to use online MICE.	
	ATT3. I think using online MICE would be enjoyable.	
	ATT4. I think using online MICE would be pleasant.	
	*Strongly disagree (1)/Strongly agree (7)*	
Behavioral intention (BI)	BI1. I expect to adopt online MICE during the COVID-19 pandemic.	(35)
	BI2. I am likely to use online MICE during the COVID-19 pandemic.	
	BI3. I have the intention to adopt online MICE in the future.	

## Results and Discussion

### Demographic Statistics

In Table 2, the proportion of male (243) and female (196) respondents was 55.4 and 44.6%, respectively. A total of 8 (1.8%) respondents were under 19 years old, 116 (26.4%) were 20–29 years old, 164 (37.4%) were 30–39 years old, 121 (27.6%) were 40–49 years old, and 30 (6.8%) were over 50 years old. In terms of education, 188 (42.8%) graduated from a university or college, 51 (11.6%) had studied at graduate school, and 168 (38.3%) had graduated from high school. The monthly average income had the following distribution: 126 respondents earning 0–4,999 yuan (40.3%), 170 earning 5,000–9,999 yuan (38.7%), 52 earning 10,000–14,999 yuan (11.8%), and 40 earning over 15,000 yuan (9.1%).

**Table 2:** Respondent demographics.

**Items**	**Category**	**Frequency**	**Percentage** **(%)**
Gender	Male	243	55.4
	Female	196	44.6
Age (years)	< 19	8	1.8
	20–29	116	26.4
	30–39	164	37.4
	40–49	121	27.6
	> 50	30	6.8
Education	Secondary school or lower	32	7.3
	High school	168	38.3
	Bachelor	188	42.8
	Postgraduate	51	11.6
Monthly income (CNY)	0–4,999	177	40.3
	5,000–9,999	170	38.7
	10,000–14,999	52	11.8
	>15,000	40	9.1

### Measurement Model Assessment

A confirmatory factor analysis was conducted to evaluate the overall model fit and the reliability and validity of the scales. The goodness-of-fit index (GFI), adjusted goodness-of-fit index (AGFI), comparative fit index (CFI), root mean square error of approximation (RMSEA), and standardized root mean square residual (SRMR) were selected to evaluate the fit of the model (Table 3) (66). The ratio of the Chi-square value to the degrees of freedom (χ^2^/df) was 1.460 (*p* < 0.05). The model fit indices (CFI = 0.977, TLI = 0.973, RMSEA = 0.032, and SRMR = 0.035) all passed their respective minimum cut-off points proposed by Hu and Bentler (67). Given the number of indicators, the overall quality of the measures was supported by the statistics (68).

**Table 3:** Confirmatory factor analysis results.

**Construct**	**Item**	**λ**	**AVE**	**CR**
PST	PST1	0.782	0.584	0.848
	PST2	0.807		
	PST3	0.746		
	PST4	0.718		
CAD	CAD1	0.720	0.570	0.841
	CAD2	0.740		
	CAD3	0.804		
	CAD4	0.753		
TRI	TRI1	0.742	0.542	0.825
	TRI2	0.721		
	TRI3	0.781		
	TRI4	0.699		
OEX	OEX1	0.800	0.573	0.843
	OEX2	0.707		
	OEX3	0.764		
	OEX4	0.753		
CTA	ATT1	0.776	0.596	0.816
	ATT2	0.779		
	ATT3	0.761		
ATT	CTA1	0.804	0.665	0.888
	CTA2	0.839		
	CTA3	0.829		
	CTA4	0.789		
BI	BI1	0.798	0.566	0.796
	BI2	0.707		
	BI3	0.750		

*Model fit indices: χ^2^/df = 1.460 (p < 0.05, df = 278); CFI = 0.977; TLI = 0.973; RMSEA = 0.032; SRMR = 0.035*.

This study uses three criteria to assess the convergent validity. First, the standardized path loading (λ) should be statistically significant and larger than 0.70 (69). Second, the composite reliability (CR) of each construct must be higher than 0.70. Third, the average variance extracted (AVE) for each construct should exceed 0.50 (70, 71). As presented in Table 3, the standardized path loadings were all significant and higher than 0.70. Moreover, the CR exceeded 0.79 for all constructs, and the AVE for each construct was >0.54. Therefore, the convergent validity for the constructs was supported.

By comparing the square root of the AVE for each construct with the correlations between the target construct and other constructs, the discriminant validity of the measurement model was determined. Discriminant validity was assumed if the square root of the AVE was larger than the correlations between the target construct and other constructs (31). The square root of the AVE for each construct exceeded the correlations between the target construct and the other constructs (Table 4), confirming the discriminant validity.

**Table 4:** AVE, correlations, and squared correlations of the constructs.

	**PST**	**CAD**	**TRI**	**OEX**	**CTA**	**ATT**	**BI**
PST	**0.584^a^**	0.245^c^	0.157	0.272	0.417	0.456	0.370
CAD	0.495^b^	**0.570**	0.116	0.209	0.372	0.282	0.187
TRI	0.396	0.340	**0.542**	0.161	0.075	0.245	0.142
OEX	0.522	0.457	0.401	**0.573**	0.197	0.356	0.277
CTA	0.646	0.610	0.273	0.444	**0.596**	0.354	0.425
ATT	0.675	0.531	0.495	0.597	0.595	**0.665**	0.366
BI	0.608	0.432	0.377	0.526	0.652	0.605	**0.566**

a *AVE values are along the main diagonal;*

b *below main diagonal lists the correlations between constructs;*

c *squared correlations between the constructs are above the main diagonal*.

### Structural Model Assessment

**Figure 2:**
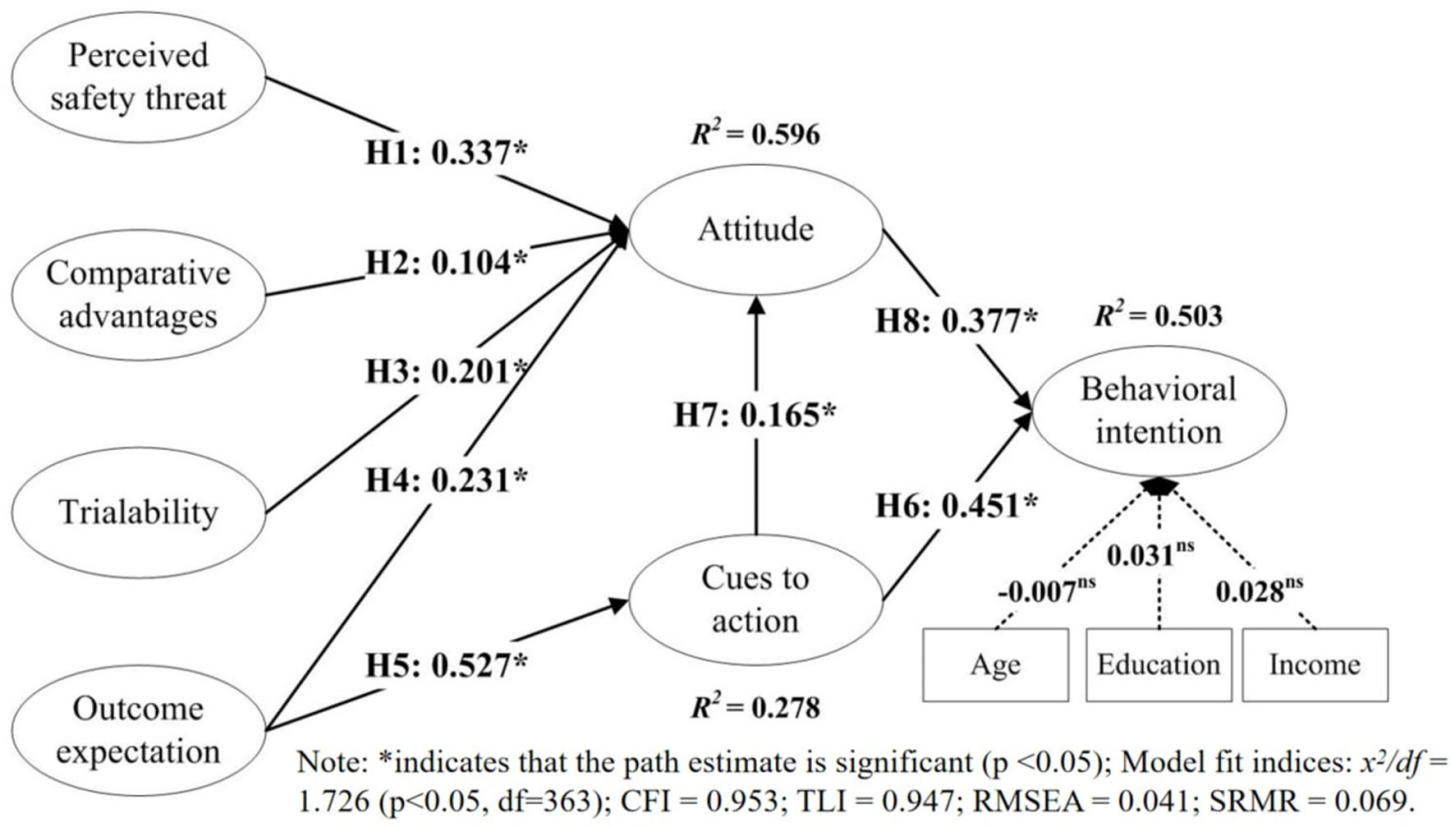
The structural model. *indicates that the path estimate is significant (*p* < 0.05); Model fit indices: *x*^2^*/df* = 1.726 (*p* < 0.05, df = 363); CFI = 0.953; TLI = 0.947; RMSEA = 0.041; SRMR = 0.069.

The hypotheses proposed in this paper were tested using a structural equation model (SEM). SEMs are often used to assess how well the structure of the proposed model or the construction of the hypotheses is explained by the collected data (72). The effect of the control variables (age, education, and income) on attendees' behavioral intention to adopt online MICE technology was determined. The reliability of experimental data was determined by a *p* < 0.05 (73). The hypotheses were tested based on the significance of the construct and the correlation of the standardized estimates. In addition, squared multiple correlations (*R*^2^) were calculated to determine the latent variables explained by the percentage variance. The results are presented in Figure 2.

Figure 2 shows that the fit of the structural model was sufficient (χ^2^*/df* = 1.726, CFI = 0.953; TLI = 0.947; RMSEA = 0.041; SRMR = 0.069). Significant positive relationships were found between attitude and the perceived safety threat, comparative advantage, trialability, and outcome expectations (0.337, 0.104, 0.104, and 0.231, respectively, *p* < 0.05). Figure 2 also shows that outcome expectations had a positive effect on cues to action (β = 0.527, *p* < 0.05). Therefore, H1 to H5 were all supported. Attendees expressed a more positive attitude toward online conference technology when they perceived greater benefits than risks. According to the HBM, the perceived safety threat reflects attendees' perception of the risks associated with attending online MICE events during the COVID-19 pandemic, whereas the outcome expectations suggest that attendees perceive net positive functional and economic benefits. The perceived comparative advantages and trialability of online MICE technology was related to attendees' attitudes toward online exhibitions, with attendees who recognize the comparative advantages of attending online MICE events and believe that online exhibition technology is feasible are more likely to hold a positive attitude toward online MICE. The positive attitudes of attendees toward online MICE technology and the perceived comparative advantages together encourage attendees to adopt online MICE technology intention instead of traditional offline MICE technology. In addition, attendee expectations about an online MICE event can influence their cues to action.

The perceived safety threat, comparative advantage, trialability, and outcome expectations explained 59.6% of the variance in attendee attitude (*R*^2^ = 0.596), and the outcome expectations explained 27.8% of the variance in cues to action (*R*^2^ = 0.278). According to the classification of *R*^2^ values by Chin et al. (74), perceived safety threat, comparative advantage, trialability, and outcome expectations had a high explanatory power for attendees' attitudes, while outcome expectation had a medium explanatory power for cues to action. These results suggest that attendees had a more positive attitude if they perceived a lower safety threat, a greater comparative advantage, high trialability, positive outcome expectations, and more positive cues to action.

The significantly positive effect of cues to action on behavioral intention (β = 0.451, *p* < 0.05) supported H6. Additionally, the correlation between cues to action and attendee attitude was 0.165 (*p* < 0.05), while attitude had a significant impact on behavioral intention (β = 0.377, *p* < 0.05); therefore, H7 and H8 were accepted. Attendees receive information about the health risks of an outbreak and recognize the benefits of following positive leads, increasing their readiness to engage in a health behavior (54). Therefore, cues to action are required for attendees to form a positive attitude toward the use of online MICE technology. In this study, cues to action and attitude were both positively correlated with the attendees' behavioral intention of online MICE (*R*^2^ = 0.503), which is consistent with the HBM. This suggests that attendees with a positive attitude are more likely to employ online MICE technology during or after the COVID-19 pandemic. This is consistent with previous studies (7, 75). Cues to action are events that guide exhibitors to exhibit safe behavior. These cues may be internal, positive, or negative experiences of exhibiting or not exhibiting safe behavior. They may also be external cues from colleagues and the media. The results showed that attendees who were given more frequent cues were more likely to engage in safe behaviors.

### Effects Analysis

To test the mediating influence of attitude on perceived safety threat, comparative advantage, trialability, and outcome expectations, behavioral intention was examined. The mediating influence of attitude on cues to action and behavioral intention was validated, and separate analyses were performed using Baron and Kenny's (76) approach. The impact of the exogenous variables on the endogenous variables was examined (Table 5). In the theoretical model presented in Figure 2, attitude fully mediates intention and the perceived safety threat, comparative advantage, trialability, and outcome expectations, while attitude partially mediates cues to action and behavioral intention.

**Table 5:** Direct, indirect, and total effects.

	**CTA (1)**	**ATT (2)**	**BI (3)**
Direct effect			-
PST (1)	-	0.337	-
CAD (2)	-	0.104	
TRI (3)	-	0.201	–
OEX (4)	0.527	0.231	-
CTA (5)	-	0.165	0.451
ATT (6)	-	-	0.377
Indirect effect			
PST (1)	-		0.127
CAD (2)	-		0.039
TRI (3)	-		0.076
OEX (4)	-	0.087	0.358
CTA (5)	-		0.062
ATT (6)	-		-
Total effect			
PST (1)	-	0.337	0.127
CAD (2)	-	0.104	0.039
TRI (3)	-	0.201	0.076
OEX (4)	0.527	0.318	0.358
CTA (5)	-	0.165	0.513
ATT (6)	-	-	0.377

In terms of the direct effects, outcome expectations had a direct effect on cues to action (a41 = 0.527). The main predictor of attitude was perceived safety threat (a12 = 0.337), followed by outcome expectations (a42 = 0.231), trialability (a32 = 0.201), cues to action (a52 = 0.165), and the comparative advantage (a22 = 0.104). Finally, cues to action had a greater direct impact on behavioral intention (a53 = 0.451) than did attitudes (a63 = 0.377).

In terms of the indirect effects, outcome expectations were the only direct exogenous variable for attitude (b42 = 0.087). The indirect influence of outcome expectations on attitude was transmitted through a single intermediary, cues to action. In addition, outcome expectations had the greatest indirect effect on behavioral intention (b43 = 0.358), followed by perceived safety threat (b13 = 0.127), trialability (b33 = 0.076), cues to action (b53 = 0.062), and the comparative advantage (b23 = 0.039). Thus, the indirect effects of the perceived security threat, comparative advantage, trialability, and outcome expectations on behavioral intention were mediated by attitude as a single mediator.

It was found that cues to action had the greatest total effect on behavioral intention (c53 = 0.513), followed by attitude (c63 = 0.377), which was traced to its direct effect on behavioral intention (a63), outcome expectation (c43 = 0.358), perceived safety threat (c13 = 0.127), trialability (c33 = 0.076), and comparative advantage (c23 = 0.039).

Finally, no statistically significant link was found between the control variables (age, education, and income) and behavioral intention, even though previous studies have shown that higher income levels, higher education levels, and people under the age of 55 have significantly higher levels of new technology and Internet access (77, 78). Regardless, this data supports the theory that theoretical concepts are more accurate predictors of attendees' behavioral intention to attend online MICE events than are demographic characteristics.

## Conclusions

### Theoretical Implications

This study makes a number of implications to existing theories. First, this study enriches the research literature on the online MICE industry in the context of COVID-19 and other public health crises. Since the end of 2019, the widespread COVID-19 pandemic has forced the convention and exhibition industry to integrate resources and has shifted offline conventions and exhibitions online, which represents a developmental opportunity for this industry. Since the emergence of COVID-19, the most of study's on the MICE industry has focused on marketing theories that explain the subsequent changes and developmental trends within the industry, while rarely analyzing consumer psychology empirically. Therefore, this research acts as an important reference for theoretical research of online MICE events.

Second, this study combines the HBM and IDT to develop a new theoretical model to understand the reasons why consumers use online MICE technology during a public health crisis. Through empirical analysis, this research found that, during COVID-19, the perceived threat and outcome expectations felt by consumers directly affected the intention to use online technology, which is also an important reason for the development of the online MICE industry. In addition, the comparative advantages and trialability of online MICE technology had an impact on consumer intentions to use the technology. This model combines consumer perception and industry characteristics to provide a comprehensive analysis of online MICE.

Third, this study enriches the HBM and IDT. Attitude and cues to action were used as intermediary variables to evaluate their influence on behavioral intention. Cues to action is an intermediary variable in the HBM, and it was verified that it plays an intermediary role between outcome expectations and behavioral intention. At the same time, attitude was proposed as an intermediary variable in the HBM and the IDT.

### Practical Implications

The findings of this study provide practical implications for MICE organizers and exhibitors in terms of enhancing their attendees' behavioral intention to attend online MICE events. First, the results indicate the need to clearly recognize that the COVID-19 pandemic poses a threat to the safety of attendees and affects how they choose to attend events. In order to overcome the security threat posed by the epidemic and to improve the outcome expectations of the attendees, MICE companies should actively innovate their exhibition strategies and extensively use new online exhibition platforms.

Second, in order to improve the comparative advantage of online exhibitions and to encourage the acceptance of online MICE technology, a digital exhibition mode is required. For example, using AR, VR, 5G technology, cloud computing, and AI technology to simulate face-to-face communication and produce targeted smart exhibition galleries could increase product sales. In addition, exhibitors can use live online broadcasts and short videos to release and promote new products online and can offer online negotiation, procurement, and docking services for exhibitors, which would not only reduce costs for exhibitors but also enable domestic and foreign companies to engage in business transactions during the COVID-19 pandemic.

Third, a positive attitude toward online MICE services will encourage attendees to participate in online MICE events. The implementation of various marketing and publicity activities on social platforms via community marketing can enhance the emotional value for customers and create a pleasant experience. This target customer may become transmitters, facilitating community fission marketing.

Finally, given the significant intermediary impact of cues to action, external cues to action such as media campaigns, alerts from health workers, and advice from others can encourage people to behave safely during the COVID-19 pandemic. The most effective way to create online MICE events is to promote positive word-of-mouth among attendees. Therefore, exhibitors should take advantage of the technical advantages of social media to segment attendee groups with different characteristics so that customers can better understand MICE brand services and that word-of-mouth marketing among attendees can be achieved.

### Limitations and Recommendations

This study has several limitations that should be considered when interpreting the results. First, this study analyzes the factors that affect attendees' behavioral intention of online MICE technology using the HBM and IDT, thus it does not consider other theoretical lenses. Future studies may thus consider incorporating the technology acceptance theory (35), planned behavior theory (79), and trust theory (32) in the model development process and to analyze the intention to attend online MICE events. With COVID-19 prevention and control in China showing a positive trend, cities across China are gradually resuming work and production, and exhibition organizers, venues, and exhibitors are also preparing for the event. Many exhibitors responded to the call for national policies and actively carried out online MICE events, which provided a good example for the digitalization of the global MICE industry. However, this study was conducted solely on a population of Chinese consumers, so the results of this study may not apply to the populations of other countries.

## Data Availability Statement

The raw data supporting the conclusions of this article will be made available by the authors, without undue reservation.

## Ethics Statement

Ethical review and approval was not required for the study on human participants in accordance with the local legislation and institutional requirements. Written informed consent from the participants' legal guardian/next of kin was not required to participate in this study in accordance with the national legislation and the institutional requirements.

## Author Contributions

JY, QP, BZ, LW, and YH equally participated in the conceptualization, literature review, data collection, data analysis, and writing of this paper. All authors contributed to the article and approved the submitted version.

## Conflict of Interest

The authors declare that the research was conducted in the absence of any commercial or financial relationships that could be construed as a potential conflict of interest.

## Publisher's Note

All claims expressed in this article are solely those of the authors and do not necessarily represent those of their affiliated organizations, or those of the publisher, the editors and the reviewers. Any product that may be evaluated in this article, or claim that may be made by its manufacturer, is not guaranteed or endorsed by the publisher.
